# Patients with Cardiac syndrome X have decreased global myocardial perfusion compared to gender matched controls; insights from CMR coronary sinus flow measurements

**DOI:** 10.1186/1532-429X-18-S1-P240

**Published:** 2016-01-27

**Authors:** Tom Gyllenhammar, Marcus Carlsson, Håkan Arheden, Henrik Engblom

**Affiliations:** 1 Department of Clinical Sciences Lund, Lund University, and Lund University Hospital, 221 85 Lund, Sweden; 2 Cardiac MR group, Dept of Clinical Physiology, Lund, Sweden

## Background

To determine if patients with Cardiac syndrome X (CSX) have decreased global myocardial perfusion during adenosine stress as assessed by cardiovascular magnetic resonance (CMR) compared to healthy controls.

## Methods

Thirteen CSX patients (64 ± 12SD years, 9 females), here defined as a history of chest pain and pathological ECG reaction during stress (> 1 mm ST-depression at stress or after 4 minutes of recovery) but normal myocardial perfusion assessed by myocardial perfusion scintigraphy (SPECT), were included. In addition, 15 positive controls (66 ± 9SD years, 6 females) with stress-induced perfusion defects by SPECT, and 18 healthy controls (44 ± 14SD years 9 females) were also included. All subjects underwent quantitative flow measurement in the coronary sinus by CMR at rest and during adenosine stress. Coronary sinus flow (ml/min) was normalized to LVM enabling quantification of global myocardial perfusion (ml/min/g). All patients underwent first-pass perfusion at rest and during adenosine stress and presence of myocardial fibrosis/infarction was assessed by late gadolinium enhancement (LGE).

## Results

**Figure 1:**
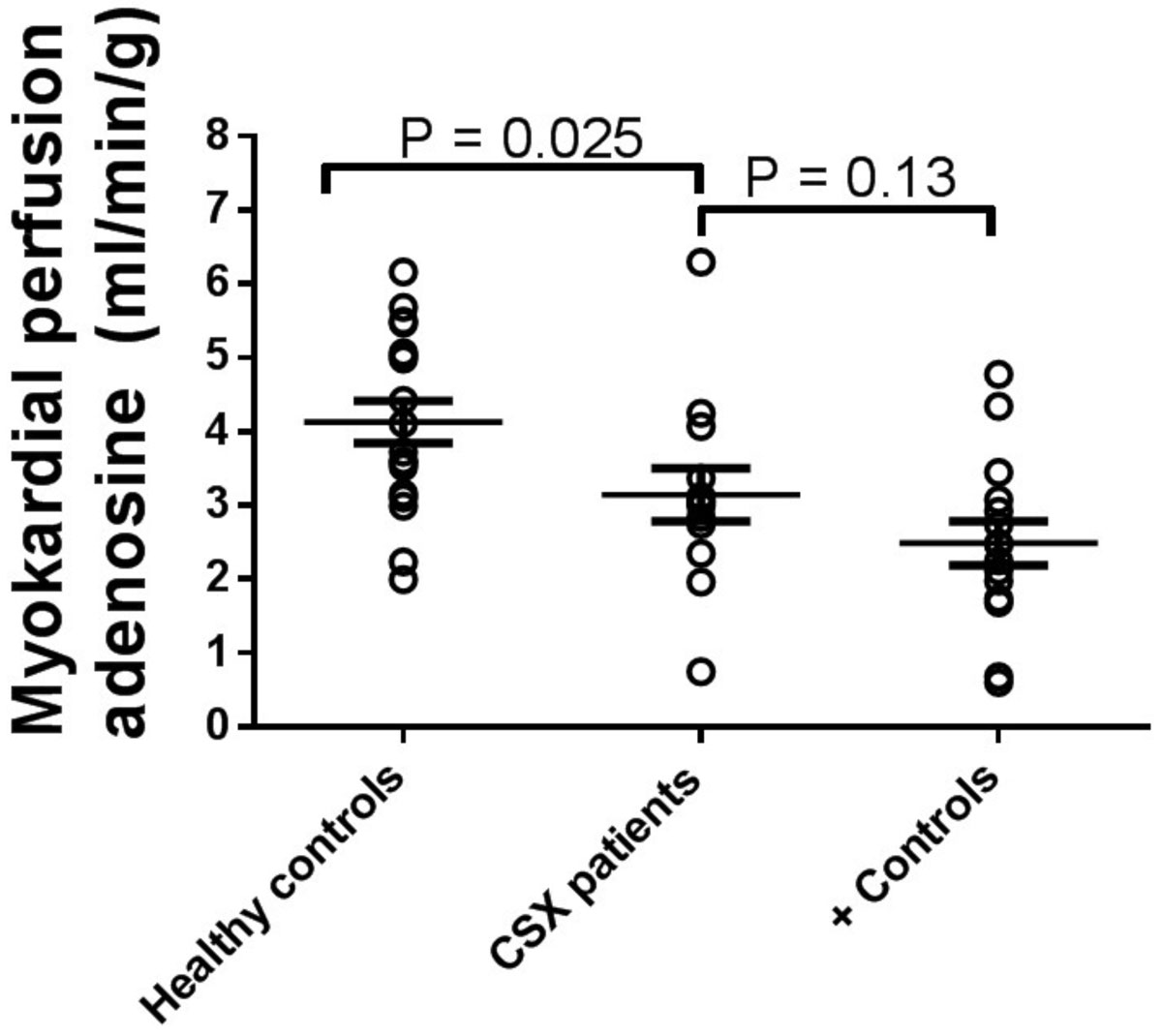
**Myocardial perfusion (MP) at rest and during administration of adenosine for each individual in the three groups**. The error bars show the mean ± SEM. MP was significantly lower during adenosine in CSX patients compared to controls, but there was no significant difference compared to positive controls.

**Figure 2:**
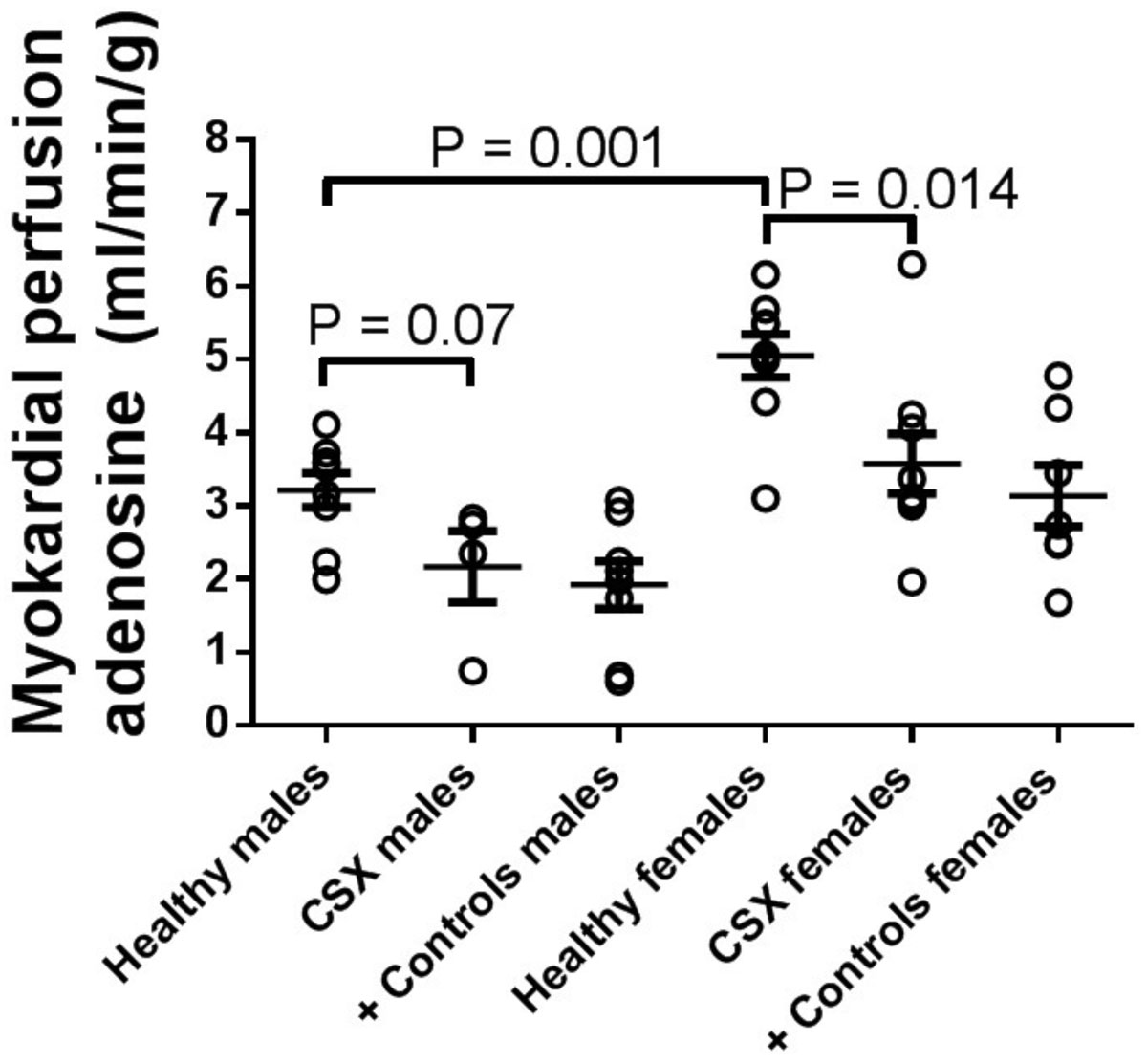
**Myocardial perfusion (MP) at rest and during administration of adenosine for each individual the three groups stratified by gender**. The error bars show the mean ± SEM. MP was significantly lower during adenosine in CSX patients compared to controls for females and a trend toward lower MP in males.

CSX patients showed significantly lower global myocardial perfusion during adenosine stress compared to healthy controls (3.1 ± 0.4 vs. 4.1 ± 0.4 ml/min/g, P = 0.025). There was, however, no significant difference between CSX patients and positive controls (2.5 ± 0.3 ml/min/g, P = 0.13; fig 1). Female controls showed significantly higher global myocardial perfusion compared to male controls (p < 0.001; fig 2). Furthermore, female CSX patients (N=9) showed significantly lower global myocardial perfusion during adenosine stress compared to female controls (3.6 ± 0.4 vs. 5.0 ± 0.3 ml/min/g, P = 0.0014). The same trend was seen for male CSX patients (N=4) compared to male controls, (2.2 ± 0.5 vs. 3.2 ± 0.2 ml/min/g, P = 0.07). No CSX patient showed myocardial fibrosis/infarction on LGE. All patients except one had normal findings on first-pass perfusion, who showed a mild regional subendocardial perfusion deficit in the inferior part of the midventricular septum.

## Conclusions

Patients with CSX, defined as history of chest discomfort and pathological stress ECG but normal regional myocardial perfusion by SPECT, have decreased global myocardial perfusion compared to healthy controls as assessed by CMR. Furthermore, there seems to be a gender difference in global myocardial perfusion. These findings indicate that CSX patients may have impaired myocardial microvascular circulation.

